# Genomic divergence in sympatry indicates strong reproductive barriers and cryptic species within *Eucalyptus salubris*

**DOI:** 10.1002/ece3.7403

**Published:** 2021-03-29

**Authors:** Rachel M. Binks, Dorothy A. Steane, Margaret Byrne

**Affiliations:** ^1^ Biodiversity and Conservation Science Department of Biodiversity, Conservation and Attractions Bentley Delivery Centre Bentley WA Australia; ^2^ School of Natural Sciences and ARC Training Centre for Forest Value University of Tasmania Hobart Tasmania Australia; ^3^ CSIRO Land and Water Sandy Bay Tasmania Australia

**Keywords:** chloroplast sequencing, cryptic speciation, dartseq, *Eucalyptus salubris*, population genomics, reproductive isolation

## Abstract

Genetic studies are increasingly detecting cryptic taxa that likely represent a significant component of global biodiversity. However, cryptic taxa are often criticized because they are typically detected serendipitously and may not receive the follow‐up study required to verify their geographic or evolutionary limits. Here, we follow‐up a study of *Eucalyptus salubris* that unexpectedly detected two divergent lineages but was not sampled sufficiently to make clear interpretations. We undertook comprehensive sampling for an independent genomic analysis (3,605 SNPs) to investigate whether the two purported lineages remain discrete genetic entities or if they intergrade throughout the species’ range. We also assessed morphological and ecological traits, and sequenced chloroplast DNA. SNP results showed strong genome‐wide divergence (*F*_ST_ = 0.252) between two discrete lineages: one dominated the north and one the southern regions of the species’ range. Within lineages, gene flow was high, with low differentiation (mean *F*_ST_ = 0.056) spanning hundreds of kilometers. In the central region, the lineages were interspersed but maintained their genomic distinctiveness: an indirect demonstration of reproductive isolation. Populations of the southern lineage exhibited significantly lower specific leaf area and occurred on soils with lower phosphorus relative to the northern lineage. Finally, two major chloroplast haplotypes were associated with each lineage but were shared between lineages in the central distribution. Together, these results suggest that these lineages have non‐contemporary origins and that ecotypic adaptive processes strengthened their divergence more recently. We conclude that these lineages warrant taxonomic recognition as separate species and provide fascinating insight into eucalypt speciation.

## Graphical abstract

Cryptic species are often detected serendipitously and don't receive thorough follow‐up investigation to verify their geographical, morphological or ecological limits. In this study, we undertake such a follow‐up with range‐wide sampling, high‐resolution population genomic (SNP) analysis, chloroplast sequencing, morphometrics and soil analysis to investigate two unexpected lineages found previously within Eucalyptus salubris. We confirm the presence of two highly divergent lineages that remain entirely genetically distinct in geographic proximity and exhibit minimal morphological separation. We conclude that historical isolation and ecotypic adaptation have likely contributed to their divergence.

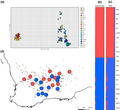

## 1 INTRODUCTION

Cryptic taxa are those that cannot be distinguished morphologically but other evidence, typically molecular, indicates that they represent different evolutionary entities (Struck et al., 2018). Such hidden lineages are increasingly being discovered across all forms of life and likely represent significant portions of unrecognized biodiversity (Bickford et al., 2006; Gill et al., 2016). Identifying cryptic divergence is necessary for gaining a more realistic appreciation of global biodiversity, informing appropriate conservation management and furthering our recognition of species boundaries beyond traditional morphological divergence (Fišer et al., 2018). However, in line with the broader difficulties surrounding species concepts and delineation approaches (e.g. de Quieroz, 2007; Galtier, 2019; Stanton et al., 2019), it remains a challenge to identify cryptic species and the processes that have led to their evolution (Fišer et al., 2018).

Speciation is typically a protracted process in which morphological, molecular, and reproductive divergence can occur to varying extents and at different rates in space and time (Queiroz, 2005, 2007). As a result of such heterogeneity, the literature abounds with numerous species concepts that cover all possible scenarios and no one concept can be widely applied to all organisms in all circumstances. However, it is a common view that reproductive isolation is fundamental to speciation and, if demonstrable, represents the strongest evidence for delineating species boundaries (Frankham et al., 2012). Such isolation can be demonstrated directly through reproductive studies (e.g. Campbell et al., 2003; Kay, 2006; Larcombe et al., 2015), indirectly by high genetic divergence under sympatric conditions (e.g. Michalski & Durka, 2015; Stuart et al., 2006) or demonstrating both factors under allopatric conditions (Frankham et al., 2012). In either case, these studies require well‐planned sampling designs to provide clear evidence of reproductive barriers. However, in the case of cryptic species, the lack of morphological variation means that divergent lineages are typically detected unintentionally while in the pursuit of other evolutionary or taxonomic information (e.g. Barrett & Freudenstein, 2011; Derieg et al., 2013; Warner et al., 2015). Consequently, cryptic taxa are often described based on limited evidence (e.g. a single genetic locus) or inappropriate sampling designs (e.g. small sample sizes) and this has led to criticism of the validity of many cryptic taxa. This is particularly true in concert with recent advances in genomic technology, where population differentiation can be mistaken for species boundaries in high‐resolution phylogenomic analyses (Sukumaran & Knowles, 2017). In this sense, detailed population genomic studies, which are well aimed at characterizing genome‐wide divergence along the population‐species continuum (Allendorf et al., 2010), are valuable as follow‐up studies to thoroughly investigate the spatial patterns of divergence across the distributions of putative cryptic taxa. This study provides such a follow‐up to investigate cryptic lineages that were unexpectedly detected within a eucalypt species.

*Eucalyptus salubris* F. Muell is the most widely distributed of nine ‘gimlet’ species that are valued for their shiny, multi‐colored fluted trunks and are endemic to the Wheatbelt and Goldfields regions of southwestern Australia (French, 2012; Johnson & Hill, 1991). Major diagnostic characters among the gimlets include the number of flowers, bud and fruit size, bud attachment and the presence or absence of glaucous branchlets (Johnson & Hill, 1991). These characters are not known to vary within *E. salubris* in any consistent manner. A previous study, designed to investigate genomic signals of adaptation in *E. salubris* along an aridity gradient, detected two highly divergent genetic lineages at opposite ends of the species’ range (Steane et al., 2015). These lineages were differentiated by both neutral and adaptive genomic markers, as well as leaf characters and their occurrence in soils of differing phosphorus levels. While intriguing, the disjunct sampling design limited an understanding of the evolutionary significance of the strong genomic divergence detected at these range extremes. Indeed, given the extensive haplotype sharing and weak reproductive barriers that are typically seen among closely related eucalypts (Grattapaglia et al., 2012; Larcombe et al., 2015), including known hybridization among the gimlet taxa (Johnson & Hill, 1991), we hypothesized that the genomic divergence observed at range extremes within *E. salubris* would likely dissolve under sympatric conditions. However, until the spatial distribution of the genomic, morphological, and ecological variation in these lineages is known, the evolutionary and taxonomic implications of the study by Steane et al. (2015) remain unclear. Given that *E. salubris* is a key species for re‐vegetation projects (French, 2012), such clarity is necessary to inform provenance sourcing for successful ecological restoration, as well as to enrich our knowledge of eucalypt diversity and speciation.

Here, we investigated the genomic, morphological, and ecological variation between the two putative lineages across the full geographic range of *E. salubris*. We had three aims based on questions stemming from the work of Steane et al. (2015). First, we assessed whether the two putative lineages represent divergence at range extremes with a genetic cline of admixture over the intervening space or whether the two lineages remain genetically distinct across the range of the species (even in sympatry), which would be indicative of reproductive isolation. To do this, we added 11 populations to the original nine sampled by Steane et al. (2015) and independently re‐assessed genome‐wide variation across the species’ full geographic range. Second, we assessed how the lineage‐associated differences in leaf morphology and soil phosphorus found by Steane et al. (2015) vary across the species’ full range. And finally, we sequenced three regions of the chloroplast genome to test for lineage‐specific haplotypes, which would be indicative of ancient lineage divergence, or in the case of no lineage‐associated haplotypes, that the two lineages result from more recent divergence within *E. salubris*. We collate these data to discuss their evolutionary implications and determine whether these lineages likely represent cryptic species or simply intraspecific variation across a wide geographic range.

## 2 MATERIALS AND METHODS

### 2.1 Study species

**FIGURE 1:**
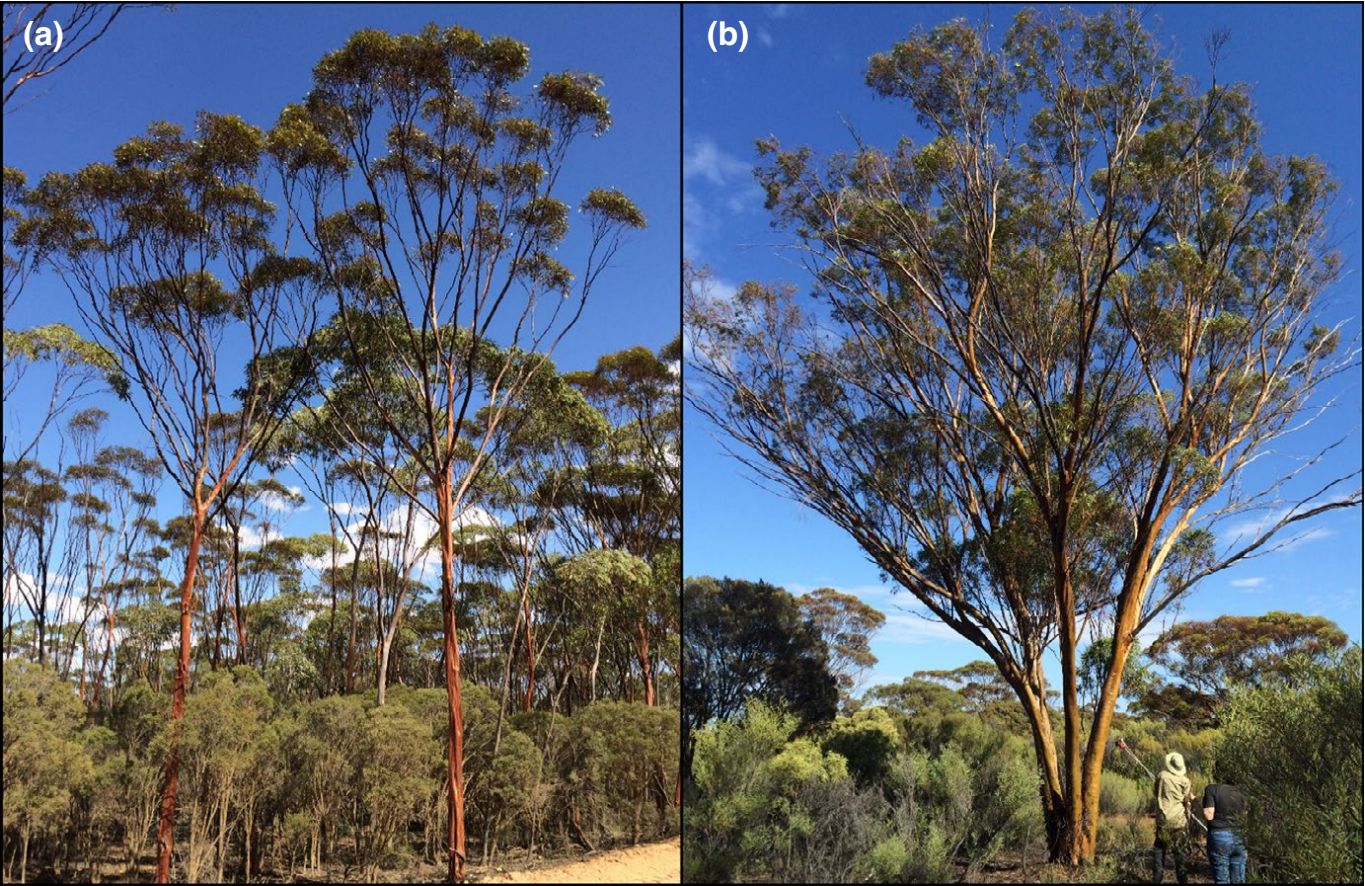
*Eucalyptus salubris* can grow as either (a) single‐stemmed or (b) multi‐stemmed trees. Note that variation in growth form is not a diagnostic character for the two cryptic lineages because both lineages can exhibit either growth form (Steane et al. 2015). Images: R Binks

*Eucalyptus salubris* is an evergreen tree up to 25 m that can grow in either single‐stemmed or multi‐stemmed form (Figure 1; Johnson & Hill, 1991). It is distributed widely across the semi‐arid to arid regions of southwestern Australia, where it forms a dominant feature of the vast, mixed eucalypt woodland that stretches across most of its range. This remote region represents an ancient landscape that remained unglaciated through the Pleistocene such that the region's flora have persisted and evolved over this long timescale (Byrne, 2007); indeed, *E. salubris* has been dated back ~1 Ma (Thornhill et al. 2019). An exception to this long historical persistence is that more recent agricultural land clearing has impacted the western edge of the species’ distribution, leaving small fragments of a once continuous population. As is typical of many large eucalypts, individuals of this species are long‐lived and can reach 400+ years in age (Gosper et al., 2013); however, the species lacks a lignotuber and mature trees are killed by fire if the whole canopy is burnt (Nicolle, 2006). While age assessment of larger individuals is challenging, *E. salubris* trees with a trunk diameter (at the base) of 15 cm have been reliably estimated to be ~100 years old (Gosper et al., 2013). The reproductive biology of *E. salubris* has not been studied specifically but the small, white flowers are likely to be generalist insect pollinated (Potts & Gore, 1995), and the seed, as with most eucalypts, simply falls to the ground beneath the tree canopy (Booth, 2017).

The two purported genetic lineages of *E. salubris* were hypothesized to be morphologically cryptic. Steane et al. (2015) found no significant difference in the number of stems, tree height, leaf size, or density between lineages but did find significant differences in leaf thickness, and in turn, specific leaf area; Lineage 1 populations were found to have significantly thinner leaves and higher specific leaf area than Lineage 2 populations. In addition, populations of Lineage 1 were found to grow on soils containing significantly higher phosphorous levels than the soils supporting Lineage 2 (Steane et al., 2015). These features are not easily applied in the field to enable straightforward identification of each lineage.

### 2.2 Sampling and DNA extraction

A total of 20 populations of *E. salubris* were sampled across the species’ distribution in southwestern Australia. Nine populations were collected in 2012 and sampling details can be found in Steane et al. (2015). In 2016, an additional 11 populations were collected in a similar manner. Given the lack of distinguishing morphological characters that could be used to identify the lineages in the field, the 11 additional populations were of unknown assignment and were selected to fill the geographic sampling gaps in the 2012 collection. Samples were taken from mature trees that were at least 10 cm in diameter at the base of the trunk (i.e. from individuals at least 50 years old; Gosper et al., 2013). Two populations of *E. ravida* were also sampled for use as an indicator of species‐level differentiation within the gimlet complex. *Eucalyptus ravida* is morphologically similar to *E. salubris* but is distinguished by conspicuously glaucous branchlets and buds (Johnson & Hill, 1991). It shares a similar evolutionary history and general ecology with *E. salubris* but has a smaller geographic distribution that is limited to the more arid, inland regions of southwestern Australia. From each of the 2016 populations, eight widely spaced trees were sampled for both genetic and morphological analysis. As with the 2012 collections, voucher specimens were taken from each 2016 population and identifications were confirmed by an expert in *Eucalyptus* taxonomy (M. French) before lodging specimens with the Western Australian Herbarium (PERTH 09209697, 09209700, 09209719, 09209727, 09209735, 09209743, 09209751, 09209778, 09209786, 09209794, 09209808, 09209816, 09209824). To provide an independent genomic analysis of the findings of Steane et al. (2015), the genomic data from the 2012 collections were discarded and the original DNA was re‐sequenced alongside the 2016 collections. To maintain consistent sample sizes across the full dataset, eight samples were chosen randomly from the 30 samples collected from each population in 2012. In total, our dataset consisted of 176 samples across 22 populations. The same DNA extraction protocol was used for both collections: a 2% CTAB method (Doyle & Doyle, 1987) was modified by adding 1% polyvinylpyrrolodine to the extraction buffer.

### 2.3 DArTseq methods and analysis

All 176 DNA samples were sent to Diversity Arrays Technology Pty Ltd (DArT, Canberra, Australia) for DArTseq™ analysis as per Sansaloni et al., (2010). Briefly, library preparation involved DNA digestion using two methylation‐sensitive restriction enzymes, *PstI* and *SphI*, and fragments were ligated with uniquely barcoded adaptors. Following PCR and quantification, the samples were standardized and pooled for sequencing in a single HiSeq 2500 (Illumina) lane. Read assembly, quality control, and SNP calling were undertaken by DArT using DArTsoft14 software, aided by alignment to the *E. grandis* genome. This pipeline uses technical replicates for a measure of genotyping reproducibility and modelled Mendelian behavior of DArTseq markers to filter sequencing errors and paralogous regions. The DArTsoft14 pipeline produced 51,897 SNP loci with 23.75% missing data. We applied further quality control filtering using the ‘*dartR’* package (Gruber et al., 2018) in *R* (R Core Development Team, 2020) to reduce the dataset to a single SNP per locus and retain only loci with reproducibility >0.95, call rate >0.90, minor allele frequency >0.02, and heterozygosity >0.02. This produced a dataset of 3,730 high‐quality SNP loci.

As a final filtering step to produce a neutral dataset for population genomic analysis, we tested for and removed any loci that may be under selection. We used BAYPASS v.2.1 (Gautier, 2015) to identify loci that represented outliers from neutral expectations and may be influenced by selection. BAYPASS utilizes an improved version of the BAYENV2 algorithm (Günther & Coop, 2013) that generates a covariance matrix to account for demographic history before using a differentiation parameter (*XtX*) to identify loci that are much more (directional selection) or less (balancing selection) differentiated than expected under neutral expectations. The *simulate.baypass* function was applied in *R* to simulate allele count data and calibrate *XtX* to determine thresholds for identifying outlier loci in the empirical datasets. We extended the running parameters (‐*nval* = 100,000, ‐*burnin* = 10,000, ‐*npilot* = 30, ‐*pilotlength* = 2,000) and across three independent runs, 125 loci were identified as outliers at the 1% threshold, representing both directional and balancing selections. These loci were removed for a final dataset of 3,605 putatively neutral loci with 2.99% missing data. This dataset was converted to program‐specific input files using PGDSPIDER v.2.1.0.0 (Lischer & Excoffier, 2012).

To investigate genetic structuring at the individual level, we first used principal coordinates analysis (PCoA) because this multivariate analysis does not rely on any particular evolutionary model (Jombart et al., 2009). PCoA was performed in *R* using the ‘*adegenet*’ package (Jombart, 2008). We then applied the Bayesian analysis implemented in FASTSTRUCTURE (Raj et al., 2014) to detect *K* genetic clusters, without any priors regarding population identity or geographic location. The simple prior was applied with the default convergence criterion, the upper *K* limit was set to 23 and the *chooseK.py* function was used to infer the most likely value(s) of *K*. Because discrete clustering patterns can be confounded with isolation by distance (Meirmans, 2012), we also used a spatially explicit alternative to FASTSTRUCTURE, as implemented in TESS v.2.3.1 (Durand et al., 2009). TESS assumes spatial autocorrelation in the data and accounts for the geographic distribution of individual samples in the clustering model. The program was run using the CAR admixture model, performing 100 iterations for each *K*‐value up to 23, with 50,000 sweeps, a burnin length of 10,000, and the default spatial interaction parameter (0.6). The most likely value(s) of *K* was determined by stabilization in the plot of the deviance information criterion (DIC) against *K*_max_.

Population differentiation (pairwise *F*_ST_) was estimated using ARLEQUIN v.35.2.2 (Excoffier et al., 2005) and visualized using the program's *R*‐lequin graphical functions. Analysis of molecular variance (AMOVA) was also performed using ARLEQUIN to partition the total genomic variation within and among the major genetic groupings detected in the above clustering analyses. Genetic diversity was assessed using the ‘*hierfstat*’ package (Goudet, 2005) in *R* to estimate allelic richness (*A*_R_) and expected heterozygosity (*H*_E_), while GENALEX v.6.501 (Peakall & Smouse, 2006) was used to calculate the percentage of polymorphic loci. To assess the relationship between genetic (*F*_ST_/(1 − *F*_ST_)) and geographic (ln(km)) distance across the whole sampled range, as well as within each of the major genetic clusters, we used mantel testing in IBDWS (available at http://ibdws.sdsu.edu/).

Finally, Bayes factor (BF) delimitation (BFD*) was used to test alternate coalescent hypotheses regarding species delimitation in our data. The analysis was run using SNAPP (Bryant et al., 2012), as implemented in BEAST v.2.5 (Bouckaert et al., 2019), to reconstruct phylogenies with alternate pre‐defined species assignments. Marginal likelihood estimates (MLE) were used to rank the alternate models, and BFs were used to determine the support for the models (Leaché *et al*., 2014). In this analysis, we tested three species models: (a) a two species model as per the current taxonomy, which considers *E. salubris* and *E. ravida* as distinct species; (b) a three species model in which we split *E. salubris* into Lineages 1 and 2, while keeping *E. ravida* a separate species; and (c) a two species model in which we split *E. salubris* into Lineages 1 and 2 but combined Lineage 2 with *E. ravida*. The last two scenarios were chosen based on the results of our population genomic analyses (Results). Because only two populations of *E. ravida* were sampled, we retained all SNP loci but randomly reduced the number of Lineage 1 and Lineage 2 samples to four to provide even representation of each potential lineage. A path sampling analysis was performed with 24 steps for each model, using one million iterations, sampling every 5,000, after a burn‐in of 1,000. The allele frequencies in the dataset were used to calculate mutation rates and default settings were applied for the priors. Convergence was assessed using Tracer v.1.7.1 (Rambaut et al., 2018) and ESS values above 200, and each model was run three times using different starting seeds to ensure consistency among runs. BFs were calculated from the MLEs following Leaché *et al*. (2014) to compare the support for Models 2 and 3, relative to the current taxonomy (Model 1).

### 2.4 Morphological and ecological traits

Of the suite of morphological, climatic, and soil traits measured by Steane et al. (2015), only specific leaf area (SLA), leaf thickness, and soil phosphorus content exhibited significant differences between the two lineages. Given that SLA and leaf thickness are strongly correlated, in the current study we measured SLA and soil phosphorus in each of the 2016 populations to add to the 2012 data and test whether these differences were maintained in line with the genomic data across the full sampled range. We applied the same methods as those detailed by Steane et al. (2015). Briefly, for SLA, 10 leaves per tree were imaged using a flatbed scanner and the area of each leaf (cm^2^) was determined in Matlab (Mathworks, USA). Leaves were dried at 55°C and weighed. Specific leaf area was calculated as the ratio of leaf area to dry mass (cm^2^/g), averaged across leaves to give a single value per tree for all *E. salubris* populations. For soil phosphorus measurements, five soil cores (0–10 cm depth) were collected across the area spanning our tree sampling within each population. The cores for each population were pooled and analyzed for nutrient content by CSBP Analytical Laboratories (Bibra Lake, Western Australia) to obtain soil phosphorus levels per population.

To test for differences in SLA and soil phosphorus between the major genetic clusters, we used *t*‐tests based on population means. Following Shapiro‐Wilk testing, the SLA dataset conformed to a normal distribution; however, the soil phosphorus data required log transformation to achieve normality. Levene's test was used to examine homogeneity of variance and the appropriate two‐tailed *t*‐test was performed for each dataset. If relevant, minor and major outliers in each dataset were identified by 1.5× interquartile range and 3× interquartile range, respectively.

### 2.5 Chloroplast sequencing and analysis

Thirteen non‐coding cpDNA regions that have been found to be useful for phylogeographic studies in Australian plants (Byrne & Hankinson, 2012) were trialed in *E. salubris*. We chose three regions for the full analysis based on sequence quality and nucleotide diversity: *rpl*16, *trn*G‐*trn*S, and *psb*D‐*trn*T. These regions were sequenced for all 176 DNA samples used in the DArTseq analysis. PCR amplifications were performed using the reaction details provided by Byrne and Hankinson (2012) and cycling conditions by Shaw et al. (2007), except 2.5 mM MgCl_2_ was used for *psbD*‐*trnT* and an annealing temperature of 52°C was used for both *trnG*‐*trnS* and *psbD*‐*trnT*. PCR products were purified using Sera‐mag SpeedBeads (Fisher Scientific, USA) and sequenced by Macrogen Inc. The relevant regions were also extracted from the whole chloroplast genome sequences of *E. salmonophloia* and *Corymbia gummifera* (GenBank Accession numbers NC_022403 and NC_022407) for use as outgroups in phylogenetic reconstruction (Bayly et al 2013, 2013). DNA sequences were aligned using ClustalW v.1.4 (Thompson et al., 1994) and concatenated in MESQUITE v.3.2 (http://mesquiteproject.org/). Any indels arising from mononucleotide repeats were ignored, while more complex indels (only found when the above outgroups were added to the alignment) were coded as single binary transitions for a total sequence length of 1,676 bp excluding the two outgroups and 1,742 bp including them.

Identification of haplotypes and calculations of haplotype (*H*_D_) and nucleotide (π) diversity were performed in DNASP v.5.10 (Librado & Rozas, 2009). We also used DNASP to calculate Tajima's *D* (Tajima, 1989) and *R*_2_ (Ramos‐Onsins & Rozas, 2002) to test for neutrality and evidence of population expansion or contraction. To test for phylogeographic structure, we calculated ordered and unordered population differentiation coefficients (*N*_ST_ and *G*_ST_, respectively) using PERMUT v.2.0 (Pons & Petit, 1996), where *N*_ST_ > *G*_ST_ indicates that haplotypes within populations are more closely related than haplotypes among populations.

To visualize the evolutionary relationships among haplotypes, we performed a maximum likelihood phylogenetic analysis using IQ‐TREE (Nguyen et al., 2015), as implemented in W‐IQ‐TREE (Trifinopoulos et al., 2016). The F81 + F + I model was determined to be the best fit for the data based on the Bayesian Information Criterion and a majority‐rule consensus tree was constructed with 1,000 ultrafast bootstrap replicates (Minh et al., 2013). We also constructed a maximum parsimony median‐joining haplotype network using NETWORK v.4.6.1.2 (Bandelt et al., 1999) because networks can be more informative than bifurcating trees in cases of limited divergence (Templeton et al., 1992), as was the case here.

## 3 RESULTS

### 3.1 DArTseq analysis

**FIGURE 2:**
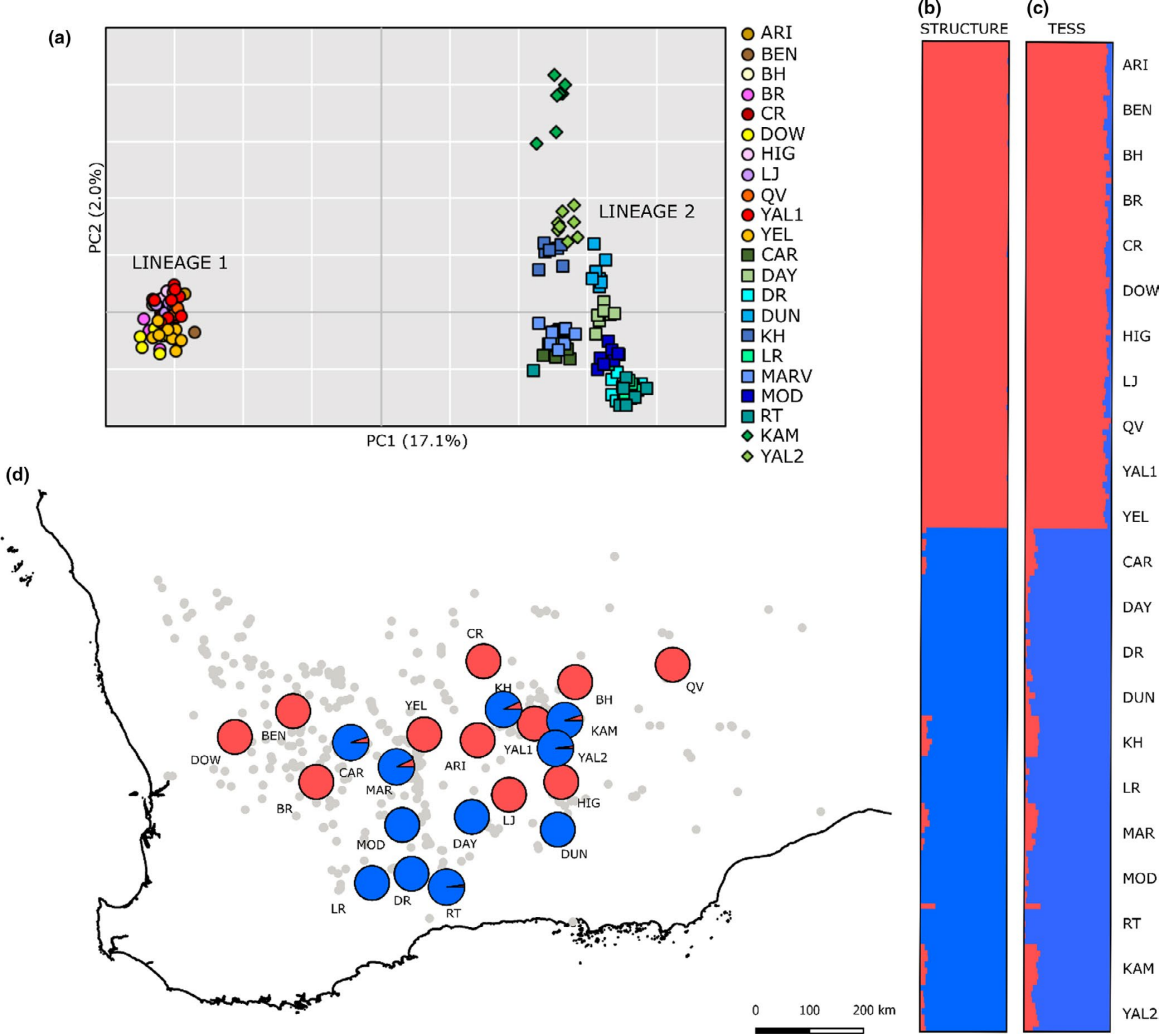
Clustering analyses of genomic variation based on 3,605 neutral SNP loci for 22 populations of *Eucalyptus salubris* and *E. ravida*. (a) Principal coordinates analysis, where populations forming Lineage 1 are represented by circles and distinguished by yellow‐red coloration, while populations forming Lineage 2 are represented by blue–green coloration, with *E. salubris* populations shown as squares and *E. ravida* populations identified by diamonds. (b) fastSTRUCTURE and (c) TESS analyses, where each individual is represented by a horizontal bar and colored according to their proportion assignment to each of two clusters that strongly associate with Lineage 1 (red) and Lineage 2 (blue). The map in (d) shows the spatial distribution of the two clusters across the sampled distribution based on the fastSTRUCTURE results. The grey dots indicate the known distribution of *E. salubris* based on records from the Western Australian Herbarium

All three clustering analyses found strong structuring in the dataset that corresponded with the two lineages found by Steane et al. (2015). The primary axis of the PCoA, which represented 17.1% of the total genetic variation, separated the data into two discrete clusters (Figure 2a) separating populations ARI, BEN, BH, BR, CR, DOW, HIG, LJ, QV, YAL1, and YEL (hereafter, Lineage 1) from populations CAR, DAY, DR, DUN, KAM, KH, LR, MARV, MOD, RT, and YAL2 (hereafter, Lineage 2). These two same groupings were also clearly identified by both FASTSTRUCTURE and TESS, where the two genetic clusters indicated minimal admixture between the lineages and populations were not mixed in assignment; all individuals within any given population were assigned to a single genetic lineage (Figure 2b,c). Geographically, Lineage 1 populations were largely in the north and Lineage 2 populations largely in the south; however, there was no clear geographical or topographical separation of the two lineages in the center of the sampled distribution, such that populations of alternate genetic lineages occurred in close proximity and yet remained genetically distinct (Figure 2d). Principal coordinate axes two (2.0%) and three (1.6%; not shown) showed minor population structuring within lineages, with tight clustering of populations in Lineage 1 but more separation of populations in Lineage 2. Interestingly, the *E. ravida* individuals were not identified as a third genetic cluster but instead were closely associated with Lineage 2 in all analyses, with some separation along PC2 in the PCoA.

**FIGURE 3:**
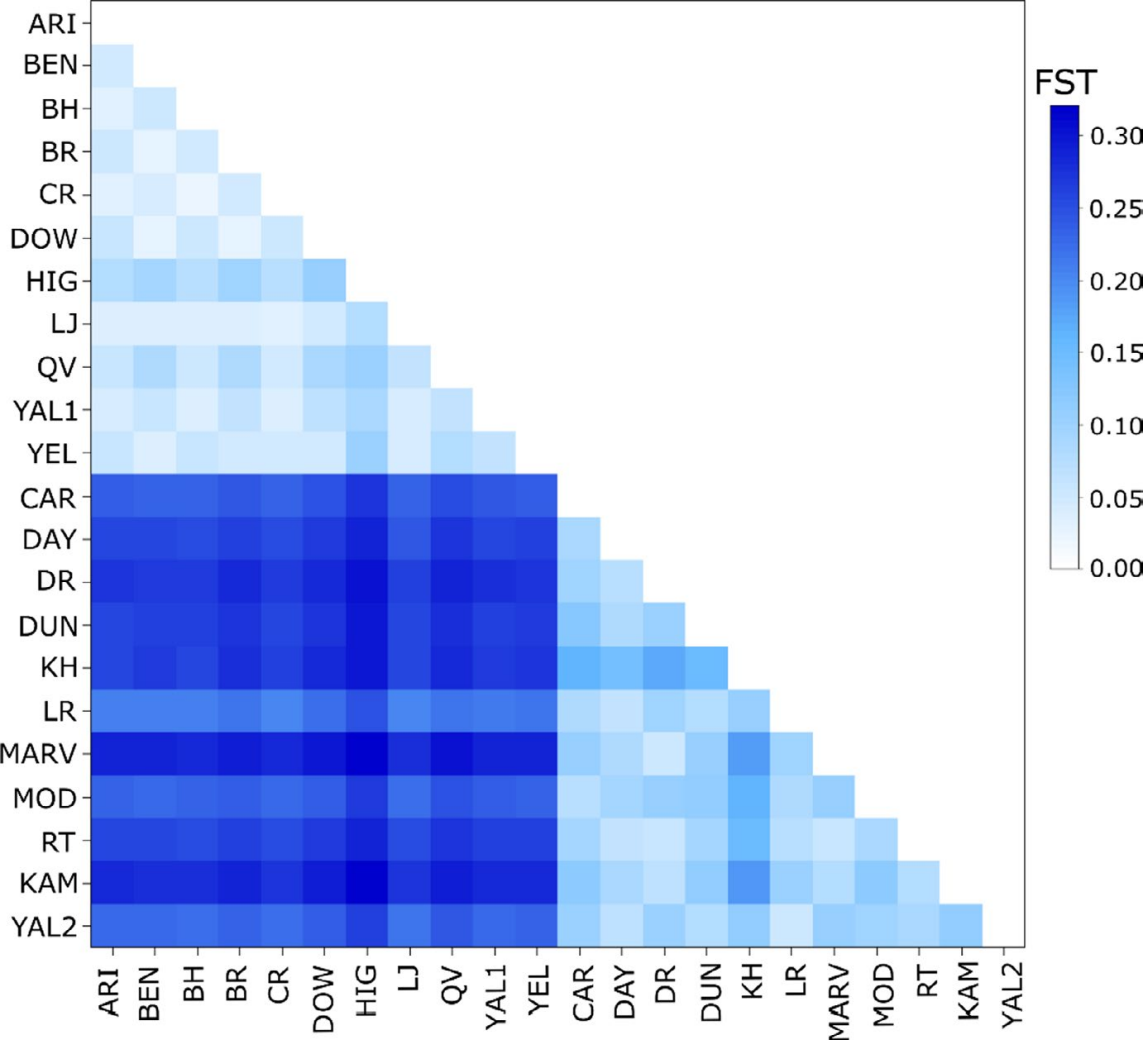
Heatmap of pairwise *F*_ST_ among 22 populations of the *Eucalyptus salubris* complex based on 3,605 neutral SNP loci. Populations are ordered by lineage to highlight the strong differentiation among lineages relative to minimal differentiation within lineages

The distinction of the two lineages was further supported by high differentiation between lineages (average *F*_ST_ = 0.252), relative to minimal differentiation within lineages (Lineage 1 *F*_ST_ = 0.046; Lineage 2 *F*_ST_ = 0.090; Figure 3). Excluding the two *E. ravida* populations, the average *F*_ST_ for Lineage 2 was reduced further to 0.067. Moreover, high levels of differentiation between populations of the two lineages were maintained irrespective of geographic proximity. AMOVA partitioned 19.67% of the total genetic variation between lineages, 6.21% among populations within lineages and 74.12% within populations. Genetic diversity was higher in Lineage 1 than in Lineage 2 across all diversity indices (Table 1). Finally, there was no correlation between genetic and geographic distance across the whole sampled range (*p* = .411) or within Lineage 2 (*p* = .450); however, there was a weak signal of isolation by distance across the range of Lineage 1 (*r* ^2^ = 0.16, *p* = .013).

**TABLE 1:** Details of sampling, including species, location and year of collection, as well as neutral SNP genomic diversity, specific leaf area, soil phosphorus content, and chloroplast haplotypes for the populations of two genetic lineages within the *Eucalyptus salubris* complex

Lineage	Species	Population (code)	Year	*%P*	*N*_A_	*H*_E_	SLA (cm^2^/g)	Soil P (mg/kg)	HAP
1	*E. salubris*	Benari (ARI)	2016	50.7	1.154 ± 0.003	0.157 ± 0.003	35.407 ± 0.536	9	H5
1	*E. salubris*	Bencubbin (BEN)	2016	49.6	1.152 ± 0.003	0.155 ± 0.003	38.919 ± 1.172	11^a^	H9
1	*E. salubris*	Bullock Holes (BH)	2012	52.2	1.159 ± 0.003	0.162 ± 0.003	39.569 ± 1.243	12^a^	H2, H3
1	*E. salubris*	Bruce Rock (BR)	2012	48.9	1.150 ± 0.003	0.153 ± 0.003	32.368 ± 0.075	7	H6
1	*E. salubris*	Credo Station (CR)	2012	51.6	1.157 ± 0.003	0.160 ± 0.003	34.737 ± 0.069	7	H3
1	*E. salubris*	Dowerin (DOW)	2016	48.5	1.151 ± 0.003	0.153 ± 0.003	36.593 ± 0.895	41^b^	H10
1	*E. salubris*	Higginsville (HIG)	2016	42.6	1.143 ± 0.003	0.145 ± 0.003	34.824 ± 0.127	4	H12
1	*E. salubris*	Lake Johnston (LJ)	2012	50.9	1.155 ± 0.003	0.158 ± 0.003	36.631 ± 0.243	5	H5
1	*E. salubris*	Queen Victoria Spring (QV)	2012	39.9	1.164 ± 0.004	0.164 ± 0.004	33.703 ± 0.119	7	H1
1	*E. salubris*	Yallari 1 (YAL1)	2016	50.4	1.155 ± 0.003	0.157 ± 0.003	34.870 ± 0.091	4	H3, H4
1	*E. salubris*	Yellowdine (YEL)	2016	48.8	1.155 ± 0.003	0.157 ± 0.003	33.140 ± 0.181	6	H3, H13, H14
MEAN ± SE				48.6 ± 1.2	1.154 ± 0.002	0.156 ± 0.002	35.524 ± 0.679	6.13 ± 0.61^c^	
2	*E. salubris*	Carrabin (CAR)	2016	45.3	1.140 ± 0.003	0.141 ± 0.003	27.116 ± 1.094	3	H3
2	*E. salubris*	Mt Day (DAY)	2016	44.5	1.136 ± 0.003	0.138 ± 0.003	28.050 ± 0.577	2	H5
2	*E. salubris*	Dunn Rock (DR)	2012	42.1	1.133 ± 0.003	0.134 ± 0.003	28.976 ± 0.490	3	H5
2	*E. salubris*	Dundas (DUN)	2016	42.2	1.134 ± 0.003	0.136 ± 0.003	27.537 ± 0.811	7^a^	H11
2	*E. salubris*	Kangaroo Hills (KH)	2012	46.8	1.146 ± 0.003	0.149 ± 0.003	29.186 ± 0.158	3	H3, H4
2	*E. salubris*	Lockhart Road (LR)	2012	40.3	1.123 ± 0.003	0.125 ± 0.003	27.895 ± 0.095	3	H5
2	*E. salubris*	Marvel Loch (MAR)	2016	44.1	1.139 ± 0.003	0.141 ± 0.003	29.861 ± 0.214	2	H3
2	*E. salubris*	Modesty Rock (MOD)	2016	44.2	1.137 ± 0.003	0.139 ± 0.003	26.238 ± 0.041	21^b^	H5
2	*E. salubris*	Ravensthorpe (RT)	2012	42	1.128 ± 0.003	0.129 ± 0.003	27.074 ± 0.126	4	H7, H8
2	*E. ravida*	Kambalda (KAM)	2016	38.6	1.133 ± 0.003	0.135 ± 0.003	n/a	n/a	H3
2	*E. ravida*	Yallari 2 (YAL2)	2016	47.1	1.144 ± 0.003	0.147 ± 0.003	n/a	n/a	H4
MEAN ± SE				43.4 ± 0.8	1.136 ± 0.002	0.138 ± 0.002	27.992 ± 0.387	2.86 ± 0.26^c^	

Abbreviations: *%P*, percent polymorphic loci; HAP, haplotype; *H*_E_, expected heterozygosity; *N_A_*, allelic richness; SLA, specific leaf area; Soil P, soil phosphorus content.

^a^ Minor outlier;

^b^ major outlier;

^c^ average calculated excluding major and minor outliers.

BF delimitation decisively rejected the current taxonomy represented by Model 1 (Table 2). The analysis returned the highest support for Model 2, with a very large positive BF value in favor of splitting the *E. salubris* lineages into two species, while retaining *E. ravida* as a third species. The marginal likelihood estimate for Model 3 was close to that for Model 2 and also strongly outperformed Model 1.

**TABLE 2:** Summary of Bayes factor delimitation (BFD) analyses, comparing three alternate species models for *Eucalyptus salubris* and *E. ravid*a

Model	Description	MLE	Rank	BF
1	Two species: *E. salubris* and *E. ravida* as per current taxonomy	₋25,122.253	3	‐
**2**	**Three species: split *E. salubris* into Lineages 1 and 2, plus *E. ravida***	₋**24,077.150**	**1**	**2,090.206**
3	Two species: split *E. salubris* into Lineages 1 and 2, but combine Lineage 2 with *E. ravida*	₋24,167.740	2	1,909.026

The model with the strongest support is indicated in bold text.

Abbreviations: BF: Bayes Factor; MLE: Marginal Likelihood Estimation.

### 3.2 Morphological and ecological diversification

Both SLA and soil phosphorus content exhibited significant differences between the two lineages across the sampled range and in a pattern consistent with Steane et al. (2015). Specific leaf area was relatively stable within lineages (Table 1) and, on average, was significantly higher in Lineage 1 populations than populations of Lineage 2 (*t* = 34.974, *df* = 18, *p* < .001). Phosphorus content was more variable across the sampled range due to inflated values in some populations of both lineages, likely due to nutrient run‐off from nearby agricultural land (Table 1). Overall, soil phosphorus was significantly higher in Lineage 1 populations than in Lineage 2 populations based on the full dataset (*t* = 2.213, *df* = 19, *p* = .039), and this difference increased following the removal of major outliers (*t* = 3.524, *df* = 17, *p* = .003) and the removal of both major and minor outliers (*t* = 5.326, *df* = 13, *p* < .001).

### 3.3 Chloroplast sequencing

All three chloroplast regions were variable and their combination resulted in 14 haplotypes across 176 samples. The three most common haplotypes, H3, H4, and H5, were shared across populations and occurred at frequencies of 23.9%, 11.4%, and 27.2%, respectively, while the remaining haplotypes were restricted to single populations and ranged in frequency from 0.6% to 4.5%. Most populations possessed a single haplotype; overall, there was an average of 1.3 ± 0.12 haplotypes per population. Nucleotide diversity and haplotype diversity across all samples were 0.002 and 0.845, respectively. Both Tajima's *D* and *R*_2_ statistics were non‐significant and thus chloroplast sequence variation is considered neutral (Table 3).

**TABLE 3:** Summary of chloroplast diversity, tests for neutrality, demographic and spatial expansion and genetic differentiation in the *Eucalyptus salubris* complex based on chloroplast sequence data

Parameter	
Sample size *n*	176
Number of haplotypes	14
Haplotype diversity	0.845
Nucleotide diversity	0.002
Tajima's *D*	−0.010
Ramos‐Onsins and Rozas *R*_2_	0.093
*N*_ST_	0.954 (0.021)*
*G*_ST_	0.902 (0.043)

* *p* < .05.

**FIGURE 4:**
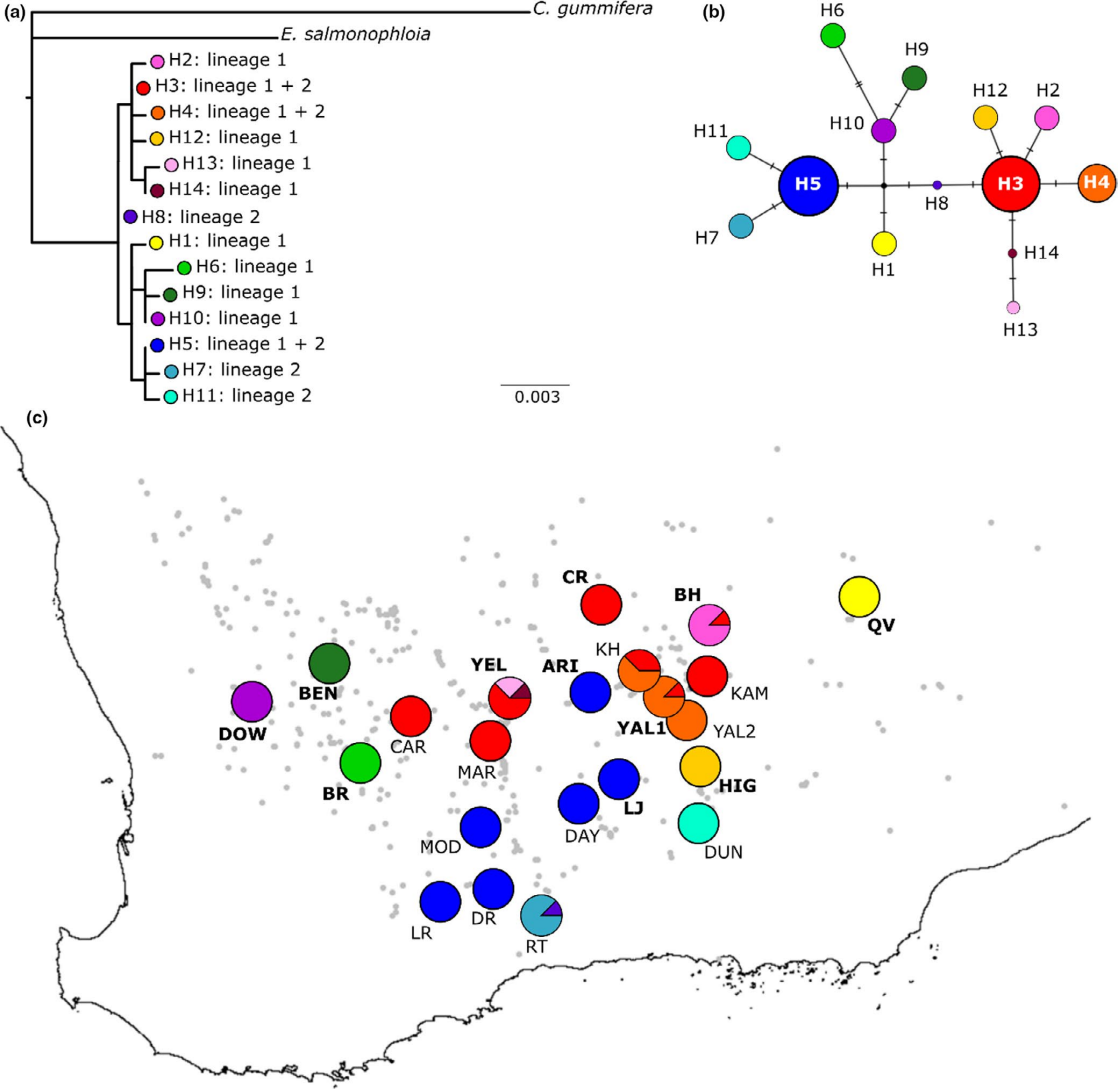
Relationships among and distribution of 14 haplotypes of three chloroplast regions (*rpl*16, *trn*G‐*trn*S, *psb*D‐*trn*T) in the *Eucalyptus salubris* complex: (a) maximum likelihood tree using *Corymbia gummifera* and *Eucalyptus salmonophloia* as outgroups (all ultrafast bootstrap support was <95%), (b) median‐joining maximum parsimony network and (c) map of haplotype distribution among populations. Lineage 1 populations are indicated with bold labels and Lineage 2 populations with non‐bold labels. The grey dots indicate the known distribution of *E. salubris* based on records from the Western Australian Herbarium

Population differentiation across the sampled range was high, and values of *N*_ST_ were significantly higher than *G*_ST_, indicating phylogeographic structure (Table 3). The maximum likelihood analysis did not resolve the relationships among haplotypes, resulting in polytomies and poor nodal support (ultrafast bootstrap support values were all <95%; Figure 4a). The relationships among haplotypes and geographic groupings were more clearly shown in the median‐joining haplotype network (Figure 4b). The network shows the two most common haplotypes, H3 and H5, separated by four substitutions, each forming a star‐like formation with a number of single‐substitution haplotypes. Additionally, a number of haplotypes (H1, H6, H9, H10) arose from an unsampled haplotype between the two major haplotypes. Geographically, haplotypes H3 and H4 dominated the northern range, H5 dominated the southern range and less common haplotypes occurred along the edges of the sampled range (Figure 4c). There was not a consistent separation of the two lineages in the chloroplast data; however, H3 and H4 were mostly associated with Lineage 1 populations in the north and H5 was mostly associated with the southern populations of Lineage 2. Inconsistencies occurred in the center of the species’ distribution, where a number of populations in Lineage 1 exhibited the southern H5 haplotype (ARI, LJ) and also in Lineage 2 populations that exhibited the northern H3 (CAR, KH, MARV) and H4 (KH, KAM, YAL2) haplotypes. This included the two *E. ravida* populations (KAM, YAL2) that associated with Lineage 2 in the SNP dataset but shared the common haplotypes, H3 and H4, found in the north. Sequence data for each haplotype have been deposited in GenBank for each chloroplast region separately (*psbD‐trnT*: MT104517‐530; *trnG‐trnS*: MT104531‐544; *rpl*16: MT104545‐558).

## 4 DISCUSSION

This study provides a comprehensive follow‐up to a previous study that unexpectedly detected cryptic lineages within *E. salubris* (Steane et al., 2015). With range‐wide sampling and an independent genomic analysis, we confirm the presence of two divergent lineages that remain strongly distinct even in geographic proximity. This presents robust, although indirect, evidence for reproductive isolation between these lineages, warranting their recognition as distinct species. In addition, we confirm that specific leaf area consistently delineates the two lineages and that their geographic distributions are associated with differing soil phosphorus levels. Finally, we find some evidence that these lineages have historical vicariant origins but were not fully differentiated until more recently. Together, these data present an intriguing case of cryptic speciation in a genus that is typically known for weak reproductive barriers.

### 4.1 Lineage divergence is maintained range wide

The additional sampling undertaken in this study was needed in order to determine whether the two lineages detected by Steane et al. (2015) represented (a) divergence in allopatry that intergrades in contact zones or (b) consistent divergence across the species’ full geographic distribution, regardless of geographic proximity. Our genomic data provide unequivocal evidence for the latter. Clear separation in each of the clustering models with no apparent admixture, combined with the marked partitioning of genetic differentiation within versus between lineages that was entirely independent of geographic distance, indicates that the distinction of these lineages is maintained in sympatry. We also confirmed that Lineage 1 dominates the northern part of the species’ range, while Lineage 2 dominates the southern part; however, the geographic boundary where the two lineages meet centrally is convoluted, presenting a mosaic of the two lineages with no obvious physical features that might restrict gene flow among populations. In eucalypts, insect, mammal, and bird pollination, in conjunction with continuous or stepping‐stone distributions, typically facilitate widespread gene flow across large distances (Byrne, 2008; Grattapaglia et al., 2012). This is reflected in the low levels of differentiation found across hundreds of kilometers within each of the two lineages, which are comparable to global *F*_ST_ values estimated with SNP datasets across similar geographic distances in other widespread eucalypt species (e.g. 0.055 in *Corymbia calophylla*: Ahrens et al., 2019; 0.017 in *E. albens* and 0.018 in *E. sideroxylon*: Murray et al., 2019; 0.079 in *E. stricta*: Rutherford et al., 2018; 0.03 in *E. regnans*: von Takach Dukai et al., 2019). Thus, pollen dispersal is not a limiting factor that could explain the abrupt genetic discontinuity that arises between adjacent populations of each lineage in the central region of our study.

Recent population formation may also explain the absence of admixture, such that this pattern in our data could simply be an artefact of the lag between recent demographic processes and the resulting signal appearing in the genome, rather than evidence of a reproductive barrier. However, comparative phylogeographic patterns throughout the sampled region have demonstrated relative environmental stability and persistence of plant populations since the Pleistocene (Byrne, 2007; Byrne et al., 2014). And while it is possible that individual populations may have extirpated and reformed in contemporary times, it seems highly unlikely that this has occurred for all populations across the central contact zone so recently as to result in zero signal of admixture across this substantial (>500 km) geographic scale. In addition, while the lineages may not co‐occur at very fine spatial scales within populations, populations of alternate lineages in the central region are geographically interspersed and occur well within the range of dispersal and gene flow, such that reproductive isolation is the most likely explanation for the maintenance of strong genomic divergence between the two lineages while in geographic proximity.

Such a case for reproductive isolation between closely related eucalypt taxa is not typical. Eucalypts are known to readily hybridize with closely related congeners within taxonomic sections (Grattapaglia et al., 2012; Griffin et al., 1988; Larcombe et al., 2015) and, indeed, hybrids have been recorded among species within the gimlet complex (Johnson & Hill, 1991). Where reproductive barriers have been studied among more divergent eucalypt species, isolation tends to be a result of both pre‐mating and post‐mating barriers (Larcombe et al., 2015). The assignment of many *E. salubris* populations to each lineage, particularly where their distributions meet, now presents an opportunity to investigate intrinsic mechanisms that may be preventing gene exchange, such as asynchrony in flowering times, gamete incompatibilities or hybrid inviability (Larcombe et al., 2015; Lowry et al., 2008). Across natural stands, specimens in the Western Australian Herbarium record *E*. *salubris* as flowering between September and March (available at www.florabase.dpaw.wa.gov.au), a wide range that may allow for two, non‐overlapping flowering seasons, one for each lineage. And while we found no genetic evidence of hybrids, our collections only sampled adult trees and post‐zygotic barriers in eucalypts often present at the juvenile stage (Larcombe et al., 2015); so it would also be interesting to explore whether hybrids exist in seed crops within natural stands of either *E. salubris* lineage. Of course, comprehensive artificial hybridization experiments would be most effective in identifying the specific mechanisms of reproductive isolation between these lineages.

### 4.2 Morphological and ecological diversification

Our range‐wide analysis also confirmed both morphological and ecological differences between the two lineages, consistent with the patterns identified by Steane et al. (2015). Populations of Lineage 1 had higher SLA, thinner leaves and occurred in soil with higher phosphorus, relative to the lower SLA, thicker leaves and lower phosphorus soil content of Lineage 2 populations. While this variation in leaf traits between lineages cannot be explained as an adaptive response to climate (Steane et al., 2015), the presence of consistent morphological differentiation between lineages, *albeit* subtle, provides an additional line of evidence for their divergence. The lack of other morphological diversifications may simply reflect yet unidentified diagnostic characters, a lag in the accumulation of morphological differences during a phase of early speciation, or perhaps selection to maintain similar morphologies across the sampled range. Furthermore, the association of each lineage with soil phosphorus levels may be indicative of ecotypic adaptation, with selection against hybrids driving divergence between the two lineages. While this association was adjusted for and remained significant following outlier removal, we acknowledge that soil phosphorus is artificially modified in some parts of the species range and, therefore, some non‐outlier values may also not reflect natural ecological variation. Nevertheless, it is worth noting that while populations of each lineage were found in close proximity, each population presented as a pure stand of a single lineage, with no detection of mixed stands. This suggests that each lineage has differential, fine‐scale environmental requirements that preclude complete sympatry. The SWAFR is known for its heterogeneous mosaic of soil profiles that are considered be a major contributor to habitat specialization and speciation of plant taxa within this biodiversity hotspot (Beard et al., 2000). Moreover, edaphic factors have been linked to cryptic divergence in other plant taxa (e.g. Martin et al., 2016; Pizano et al., 2011; Yost et al., 2012), and phosphorus, specifically, has been demonstrated to have differential impacts on seedling survival and growth of other eucalypt species (*E. regnans*: Ashton & Kelliher, 1996; *E. grandis*: Tng et al., 2014). Whether it be phosphorus or another soil component correlated with phosphorus, it is quite feasible that edaphic adaptation of the *E. salubris* lineages has presented a strong extrinsic barrier to gene flow, rather than, or in addition to, intrinsic reproductive barriers. Testing of this hypothesis would require reciprocal transplant experiments assessing the germination, growth, and survival of seedlings under differing soil conditions.

### 4.3 Historical origins but more recent resolution

The phylogeographic structure and uneven distribution of haplotypes across the sampled range are indicative of a history of isolation and divergence within *E. salubris*. This is a common feature of widespread plant species in the SWAFR (e.g. Byrne & Hines, 2004; Byrne et al., 2002; Nistelberger et al., 2014; Wheeler & Byrne, 2006), where historical climate fluctuations are thought to have driven broad contraction to general refugia during periods of high aridity in the mid‐Pleistocene resulting in highly divergent lineages (Byrne, 2007; Byrne et al., 2014). In our dataset, the north‐south spatial segregation of the two main but divergent haplotypes (H3 + H5, respectively) and the occurrence of unique haplotypes in edge populations are consistent with a pattern of contraction to the range extremes with localized persistence. Moreover, the higher haplotype diversity in the north suggests that northern populations remained relatively large and stable, while the small hotspot of diversity in the southernmost populations around the Ravensthorpe Range may be indicative of a southern refugium to which the species contracted, followed by subsequent northward re‐expansion of the H5 haplotype and secondary contact with the northern region. The Ravensthorpe Range is a center of diversity (Hopkins et al., 1983; Hopper et al., 1996) and is known to harbor divergent haplotypes in other species (Byrne et al., 2001). Clearly, these northern and southern haplotypes show broad geographic associations in line with the SNP dataset and it is likely that historical isolation and drift have played a role in the divergence of the two lineages.

However, in contrast to the strong divergence seen in the SNP dataset, the overall level of divergence in the chloroplast data was low and the haplotypes in the central region did not consistently delineate each lineage. Haplotype sharing between the SNP lineages may be indicative of historically incomplete lineage sorting or incomplete reproductive barriers. And from a general plant perspective, haplotype sharing might negate evidence for divergence but in the specific context of eucalypt evolution, where haplotype sharing is very common among species due to reticulate evolution, chloroplast capture, and incomplete lineage sorting (e.g. McKinnon et al., 2001, 2004; Nevill et al., 2014; Steane et al., 1998), it is not surprising to see some haplotype sharing in our dataset. Rather, in that context, the existence of lineage‐specific haplotypes presents robust evidence that lineage divergence has not arisen in recent generations but has developed over a long time period. It follows that isolation and drift may have initiated divergence some time ago, and ecotypic adaptation to differing edaphic conditions has driven further divergence and strengthened reproductive isolation in the present‐day lineages.

### 4.4 Taxonomic implications

The substantial and geographically abrupt genomic differentiation observed between the two lineages of *E. salubris* is indicative of strong reproductive isolation. In conjunction with the clear rejection of the current taxonomic model by the BFD* analysis that showed the ‘three species’ model to be superior, we suggest that these two lineages warrant taxonomic recognition as separate species. This reasoning specifically aligns with the Biological Species Concept (de Queiroz, 2007) but follows the underlying theory of most species’ concepts (Frankham et al., 2012). From a comparative perspective, the fact that the genomic differentiation between lineages is greater than that between Lineage 2 and *E. ravida* provides further evidence supporting the recognition of these lineages at the species level. Given that the type specimen of *E. salubris* was collected in the far north of the species’ range (Johnson & Hill, 1991), it follows that Lineage 1 should remain *E. salubris* and Lineage 2 should be re‐named. Unfortunately, these taxa remain cryptic because SLA is not readily observed or measured in the field. Our finding of significant morphological and ecological differences suggests that more diagnostic features may be exposed with further investigation. Now that a number of populations have been attributed to each lineage, it would be worthwhile revisiting these sites and thoroughly assessing each taxon for other morphological characters that may be more useful for field identification, as well as flowering time.

The association of *E. ravida* with Lineage 2 was unexpected given their morphological distinction and current taxonomic recognition as separate species. However, this result is more in line with studies of other closely related eucalypt species, where distinct morphological differentiation is often not reflected by strong nuclear genetic differentiation (e.g. Pollock et al., 2013; Rutherford et al., 2018; Shepherd & Raymond, 2010). While Lineage 2 is clearly a distinct species from Lineage 1, it is not yet clear whether Lineage 2 represents a new species or is a variant of *E. ravida*. Indeed, while the ‘three species’ model had the highest support in the BFD* analysis, the ‘two species’ model combining Lineage 2 with *E. ravida* was also supported over the current taxonomy, indicating some uncertainty in the distinction of these two taxa. Thus, before taxonomic changes regarding Lineage 2 can be made, further investigation with greater sampling of *E. ravida* is needed to resolve the relationship between Lineage 2 and *E. ravida*.

Under both scenarios, our results strongly indicate that cryptic speciation has occurred within the gimlet complex. In the case that Lineage 2 and *E. ravida* are different species, as per the best‐supported BFD* model, Lineages 1 and 2 are clearly established as separate species that are morphologically cryptic from each other (other than SLA, which is not readily observable in the field). Alternatively, the only recorded morphological difference between both lineages of *E. salubris* and *E. ravida* is glaucousness (Johnson & Hill, 1991) so in the case of the second best‐supported BFD* model where Lineage 2 and *E. ravida* are the same species, glaucousness is no longer a diagnostic character and that leaves the morphological comparison of Lineage 1 versus Lineage2/*E. ravida* as still being cryptic. Given the complexity of divergence seen both within *E. salubris* and between *E. salubris* and *E. ravida*, any future investigation of this study system would benefit from the molecular examination of all nine taxa currently recognized within the gimlet complex. Furthermore, given that some of these taxa are known to hybridize (Johnson & Hill, 1991) and that eucalypt divergence in the nuclear genome is often not matched in the plastid genome due to haplotype sharing (e.g. Grattapaglia et al., 2012; Healey et al., 2018; Schuster et al., 2018), we suggest that such an investigation would yield greater clarity of evolutionary relationships using genomic data from the nuclear genome, rather than the chloroplast genome.

## 5 CONCLUSIONS

In our comprehensive analysis of populations across the geographic range, we demonstrate that substantial genomic divergence is maintained between two previously detected lineages of *E. salubris*. In addition, these lineages are strongly associated with differences in specific leaf area and soil phosphorus content. Finding genomic, morphological, and ecological evidence of divergence that is maintained across the extensive zone of geographic proximity is strong evidence for reproductive isolation and we suggest that these lineages warrant recognition at the species level. Future work could focus on identifying the mechanism of reproductive isolation and a broader phylogenomic investigation of the whole gimlet complex. Finally, the existence of these cryptic taxa needs be made known to managers actively working with *E. salubris* in ecological restoration activities because unknowingly mixing or translocating these taxa outside of their preferred niches may limit success.

## CONFLICT OF INTEREST

We have no conflicts of interest to declare.

## AUTHOR CONTRIBUTION

**Rachel Maria Binks:** Data curation (lead); Formal analysis (lead); Methodology (lead); Writing‐original draft (lead). **Dorothy Steane:** Conceptualization (equal); Data curation (supporting); Writing‐review & editing (supporting). **Margaret Byrne:** Conceptualization (equal); Funding acquisition (lead); Project administration (lead); Supervision (lead); Writing‐review & editing (supporting).

## ETHICAL APRROVAL

We have no ethical considerations to declare.

## Data Availability

Voucher specimens have been lodged at the Western Australian Herbarium (PERTH 08593760, 08593779, 08593787, 08593795, 08593809, 08593817, 08593825, 08593833, 08593841, 08593868, 08593876, 08593884, 09209697, 09209700, 09209719, 09209727, 09209735, 09209743, 09209751, 09209778, 09209786, 09209794, 09209808, 09209816, 09209824). Data regarding SNP genotypes, specific leaf area, and soil phosphorus content are available on Dryad (doi.org/10.5061/dryad.b8gtht7bx), and chloroplast sequence data are available on GenBank (MT104517‐558).
